# BDS-Adam optimizer integrating adaptive variance rectification with semi-adaptive gradient smoothing

**DOI:** 10.1038/s41598-025-20788-y

**Published:** 2025-10-22

**Authors:** Yichuan Shao, Shiqian Weng, Haijing Sun, Qian Gao, Le Zhang, Zhiqiang Mao, Shuai Xu, Zhitao Zhang, Lei Xing

**Affiliations:** 1 School of Intelligent Science and Information Engineering, Shenyang University, Shenyang, 110044 China; 2 Key Laboratory of Computing Power Network and Information Security, Shandong Computer Science Center (National Supercomputer Center in Jinan), Ministry of Education, Qilu University of Technology (Shandong Academy of Sciences), Jinan, 250014 China; 3 Shandong Provincial Key Laboratory of Industrial Network and Information System Security, Shandong Fundamental Research Center for Computer Science, 250014 Jinan, China; 4 College of Intelligent System Science and Engineering, Laboratory of Intelligent Manufacturing and Micro-Nano Processing, Shenyang University, Shenyang, 110044 China; 5 No.87 Danan Street, Shenhe District, Shenyang, Liaoning province China; 6 Shenyang Women’s and Children’s Hospital, Shenyang, 110011 China; 7 School of Chemistry and Chemical Engineering, University of Surrey, Guildford, GU2 7XH UK

**Keywords:** Adaptive optimizer, Variance correction, Gradient smoothing, Deep learning, Convergence analysis, Engineering, Mathematics and computing

## Abstract

In this work, an enhanced variant of the Adam optimizer, termed BDS-Adam, is proposed to address two critical limitations of the original Adam algorithm: biased gradient estimation and training instability during early optimization. To overcome these issues, a dual-path framework is adopted. In the first path, a nonlinear gradient mapping module (adaptive reshaping of raw gradients using hyperbolic tangent) is applied to adaptively reshape raw gradients, enabling the optimizer to better capture local geometric structures. In the second path, a semi-adaptive gradient smoothing controller–based on real-time gradient variance–is incorporated to suppress abrupt parameter updates and stabilize training dynamics. These two outputs are integrated through a gradient fusion mechanism (combining smoothed and transformed gradients before updates), in which smoothed and transformed gradients are combined prior to parameter updates. Moreover, an adaptive second-order moment correction technique is employed to mitigate cold-start effects caused by inaccurate variance estimates in the early training phase. A convergence analysis under non-convex settings is provided, and it is theoretically demonstrated that the expected gradient norm is bounded under standard assumptions, indicating improved robustness and long-term stability. This adaptive bias-correction formulation further improves training stability. Empirical evaluations on three benchmark datasets–CIFAR-10, MNIST, and a gastric pathology image dataset–reveal test accuracy improvements of 9.27%, 0.08%, and 3.00%, respectively, compared to Adam. These results confirm that the proposed dual-mechanism optimizer effectively enhances both convergence speed and generalization performance across diverse tasks.

## Introduction

Optimization algorithms have been recognized as crucial components in the training of deep neural networks, especially in complex tasks such as image classification. Among these, Adam^1^ and its numerous variants have been widely adopted due to their ability to combine momentum with adaptive learning rates, which often accelerate convergence and improve training stability. However, several inherent limitations of Adam have been identified. Specifically, biased gradient estimation, unstable training during early iterations (commonly referred to as cold-start issues), and sensitivity to gradient variance and hyperparameter settings have been observed^2,3^. These challenges are particularly pronounced in non-convex optimization landscapes, where inaccurate moment estimates^2,4^ and noisy gradients can cause oscillations and slow convergence^1,2,5,6^.

BDS-Adam fundamentally resolves the non-convergence flaw inherent in adaptive moment estimators.

To address these limitations, various Adam-based optimization strategies have been introduced. For instance, AMSGrad^7^ was proposed to ensure convergence by modifying the second-order moment update rule. AdaBound^8^ and AMSBound introduced dynamic learning rate bounds to improve generalization. Padam^9^ and AdamSSM^10^ integrated projection and second-order system dynamics, respectively. Nevertheless, most of these methods retain static or heuristic control structures, and insufficient attention has been paid to mitigating gradient noise and addressing instability during early training stages.

BDS-Adam mitigates the non-vanishing gradient bias issue of Adam^2^ by incorporating Borgesian Gradient-Aware Smoothing and adaptive variance rectification, thereby improving stability and convergence. Comparative analysis with dominant variants reveals: AMSGrad ensures convergence but lacks noise-adaptive smoothing, failing to suppress cold-start oscillations; Radam^11^ improves stability via symplectic correction at the cost of quadratic computational complexity; BDS-Adam uniquely integrates semi-adaptive smoothing with gradient fusion, accelerating convergence while preserving linear computational complexity $\:\left(\mathcal{O}\left(d\right)\right)\:$for BDS-Adam and Adam, versus ($\:\mathcal{O}\left({d}^{2}\right)$) for RAdam under the same dimensionality $\:d$.

In addition to gradient-based optimizers, hybrid and population-based optimization methods have been proposed. For instance, Brezinski and Ferens introduced a cognitive hybrid Particle Swarm Optimization/Simulated Annealing (PSO/SA) approach for combinatorial optimization problems^12^. Similarly, Abed-alguni (2019) proposed the island-based Cuckoo Search with polynomial mutation (iCSPM), where iCSPM denotes island-based Cuckoo Search with polynomial mutation^13^, and H. & PaulDavid (2022) introduced an enhanced version (iCSPM2), where iCSPM2 denotes iCSPM with elite opposition-based learning^14^. Other extensions include the original Cuckoo Search algorithm by Yang and Deb^15^, a cuckoo search variant with adaptive discovery probability based on double Mersenne numbers^16^ and the Improved Salp Swarm Algorithm with Hierarchical Dynamic Parameter Mechanism (ISSA-HDPM)^17^. Moreover, multi-objective PSO has been applied in classification and feature selection tasks^18^, demonstrating the broader utility of population-based optimizers. While these techniques exhibit promising performance in low-dimensional or discrete domains, their applicability to high-dimensional deep learning problems remains limited due to high computational costs and incompatibility with continuous gradient updates.

Beyond these methods, several more recent Adam variants and hybrid optimizers further expand the optimization landscape^19^. Lookahead introduces a slow-weight update mechanism that stabilizes the trajectory of fast inner updates, thereby improving robustness across tasks^20^. AdaBelief refines variance adaptation by considering the “belief” in the gradient direction, combining Adam-like fast convergence with SGD-like generalization^21^. AdaBound imposes dynamic bounds on learning rates, enabling a smooth transition between adaptive and non-adaptive behavior^8^. LAMB achieves scalable large-batch training through layer-wise adaptive learning rates, making it suitable for training very large models such as BERT^22^. In addition, hybrid and alternative schemes such as Ranger (RAdam + Lookahead), COCOB and coin-betting approaches, stochastic line-search methods, and communication-efficient variants like 1-bit LAMB^23^ highlight the diversity of optimizer design^24,25^. These works collectively underscore the importance of addressing gradient noise, stability, and scalability, and they provide a broader landscape against which BDS-Adam can be positioned.

To explicitly address the instability and cold-start limitations of Adam, a novel optimizer named BDS-Adam is proposed in this study. It integrates two key mechanisms: (1) a nonlinear gradient mapping module^26^ that adaptively transforms raw gradients to better capture local geometric curvature, and (2) an adaptive momentum smoothing controller that dynamically adjusts momentum coefficients in response to real-time gradient variance^27^. These two outputs are integrated through a gradient fusion mechanism, producing stable and geometry-aware parameter updates. Additionally, an adaptive second-order moment correction technique is adopted to mitigate cold-start effects caused by biased variance estimates in early iterations, thereby enhancing training robustness.

Theoretical analysis demonstrates that BDS-Adam satisfies the convergence condition of bounded expected gradient norms in non-convex settings under standard assumptions. Moreover, under identical Lipschitz smoothness and bounded variance assumptions, BDS-Adam achieves a convergence rate that matches or surpasses Adam, AMSGrad, and RAdam, while maintaining the same order of computational complexity as Adam, thereby highlighting its theoretical soundness and practical efficiency.

The main contributions of this work are summarized as follows:

- A Dual-Path Optimization Framework: Incorporating nonlinear gradient mapping and adaptive momentum smoothing into a unified architecture.
- Adaptive Variance Correction: A bias correction mechanism that stabilizes training under cold-start conditions.
- Gradient Fusion Mechanism: Integrating dual-path outputs to generate stable and geometry-aware updates.
- General Applicability: The optimizer is validated across diverse datasets and demonstrates superior performance compared to both classical and state-of-the-art Adam variants.

## Results

In this study, an improved optimization algorithm named BDS-Adam was developed to address the challenges of biased gradient estimation and early-stage training instability, both of which are known limitations of the standard Adam optimizer. The core innovation lies in a dual-path architecture that integrates two key mechanisms: an adaptive variance correction module and an adaptive momentum smoothing controller.

In the first path, a nonlinear gradient mapping module, utilizing a hyperbolic tangent function, was employed to adaptively reshape the historical gradient retention ratio. This adaptation allows for enhanced sensitivity to local curvature. In the second path, the adaptive momentum smoothing controller was designed to dynamically adjust momentum coefficients based on real-time gradient variance, effectively suppressing abrupt parameter updates and improving training robustness. These two outputs were combined through a gradient fusion mechanism, resulting in stable and geometry-aware parameter updates. Furthermore, an adaptive second-order moment correction was introduced to mitigate cold-start effects and accelerate early-stage convergence.

Empirical evaluations on CIFAR-10, MNIST, and a gastric pathology dataset demonstrated test accuracy improvements of 9.27%, 0.08%, and 3.00%, respectively. All experiments were conducted under consistent model architectures to eliminate confounding factors. Across all datasets, BDS-Adam exhibited improved gradient stability, faster convergence, and favorable performance compared to classical optimizers such as SGD, AdamW, Adagrad, and NAdam. Additionally, BDS-Adam showed a near-Pareto balance between accuracy and convergence speed, without requiring additional computational overhead.

Despite its advantages, BDS-Adam has some limitations. It introduces additional hyperparameters—such as the smoothing coefficient and gradient normalization scale—which may require task-specific tuning, and its generalizability to large-scale, multimodal, or non-visual domains (e.g., natural language processing or reinforcement learning) remains to be evaluated. Furthermore, although stability has been verified in pathological curvature regions via local quadratic approximation, global convergence guarantees under highly sparse gradients or extreme curvature conditions require further investigation. Future work will include formal convergence rate analysis and systematic hyperparameter sensitivity studies to enhance both theoretical understanding and practical robustness in broader applications.

### BDSAdam algorithm design

#### Adam optimization algorithm

Adam (Adaptive Moment Estimation) is an optimization algorithm that combines momentum with an adaptive learning rate mechanism^1^. It estimates the first-order (mean) and second-order (uncentered variance) moments of the gradients by computing the exponential moving averages of the gradient and its square, respectively. These statistics are then used to adaptively adjust the learning rate of each parameter, thereby addressing the challenges of fixed learning rates in traditional stochastic gradient descent and improving convergence speed.

The moment estimation process is defined as follows:

1 $$\:\begin{array}{c}{m}_{t}={\beta\:}_{1}{m}_{t-1}+\left(1-{\beta\:}_{1}\right){g}_{t}\end{array}$$

2 $$\:\begin{array}{c}{v}_{t}={\beta\:}_{2}{v}_{t-1}+\left(1-{\beta\:}_{2}\right){g}_{t}^{2}\end{array}$$

where $\:{m}_{t}$ and $\:{v}_{t}$ represent the biased first-order and second-order moment estimates at time step $\:t$, $\:{\beta\:}_{1}$,$\:{\beta\:}_{2}$∈ (0, 1) are the exponential decay rates for the respective moment estimates, and $\:{g}_{t}$ is the gradient at the current iteration.

To mitigate the initialization bias caused by zero initialization of moment vectors, bias correction is applied as follows^1,2^:

3 $$\:\begin{array}{c}\stackrel{\wedge\:}{{m}_{t}}=\frac{{m}_{t}}{1-{\beta\:}_{1}^{t}}\end{array}$$

4 $$\:\begin{array}{c}\stackrel{\wedge\:}{{v}_{t}}=\frac{{v}_{t}}{1-{\beta\:}_{2}^{t}}\end{array}$$

The parameters are then updated using the corrected moments:

5 $$\:\begin{array}{c}{\theta\:}_{t+1}={\theta\:}_{t}-\frac{\eta\:}{\sqrt{\stackrel{\wedge\:}{{v}_{t}}}+\varepsilon\:}\stackrel{\wedge\:}{{m}_{t}}\end{array}$$

where $\:\eta\:$ denotes the global learning rate, and $\:\varepsilon\:$ is a small constant added to improve numerical stability and avoid division by zero.

Adam has demonstrated improved convergence speed and adaptability in many deep learning applications. However, its performance is sensitive to hyperparameter settings and it may converge to suboptimal solutions in some cases^28^, which can affect model robustness and generalization performance^6^.

## Semi-adaptive normalized gradient smoothing

Among traditional gradient smoothing methods, Exponential Moving Average (EMA) has been widely adopted due to its simplicity^29,30^. However, its use of fixed smoothing factors prevents it from effectively distinguishing between noisy gradients and meaningful update directions. In particular, over-smoothing often occurs in flat regions of the loss surface, while delayed responses are observed in regions where gradients change abruptly.

To address these limitations, an adaptive momentum smoothing controller was introduced in this study, inspired by the Borges smoothing principle. This controller dynamically adjusts the smoothing factor based on the L2 norm of the current gradient, thereby enabling noise suppression when the gradient is small, and responsive updates when the gradient is large.

The traditional EMA formulation is defined as:

6 $$\:\begin{array}{c}{g}_{smooth}^{\left(t\right)}=\beta\:{g}_{smooth}^{\left(t-1\right)}+\left(1-\beta\:\right){g}^{\left(t\right)}\end{array}$$

The formula $\:\beta\:$ is a fixed smoothing coefficient.

Where $\:{\upbeta\:}$ is a fixed smoothing coefficient.

In contrast, the proposed mechanism replaces the fixed factor $\:{\upbeta\:}$ with a semi-adaptive gradient smoothing factor $\:{\lambda\:}_{t}$, defined as:

7 $$\:\begin{array}{c}{\lambda\:}_{t}=\lambda\:(1-{tan}h(\|{g}_{t}\|))\end{array}$$

where $\:\lambda\:$ is the base smoothing factor (set as semi-adaptive smoothing in this study),$\:\|{g}_{t}\|$ denotes the L2 norm of the current gradient, the tanh function compresses the gradient magnitude into the interval [0,1], ensuring a smooth response to gradient changes.

The dynamic characteristics of this mechanism can be summarized as:

When $\:\|{g}_{t}\|$ → ∞,$\:{tanh}(\|{g}_{t}\|)$ → 1, and $\:{\lambda\:}_{t}\approx\:0$: smoothing is minimized, allowing the optimizer to rapidly respond to sharp gradients.

When $\:\|{g}_{t}\|$ → 0,$\:{tanh}(\|{g}_{t}\|)\to\:0$, and $\:{\lambda\:}_{t}\approx\:\lambda\:$: smoothing is enhanced, suppressing noise during early-stage training.

To ensure numerical stability and prevent extreme values of $\:{\lambda\:}_{t}$, the coefficient is constrained within the interval [0.1,0.9]:

8 $$\:\begin{array}{c}{\lambda\:}_{t}=clamp\left({\lambda\:}_{t},\text{0.1,0.9}\right)\end{array}$$

In the exponential moving average process, the effective memory length $\:{N}_{\text{e}\text{f}\text{f}}$-defined as the number of steps over which historical gradients exert a significant influence on the current smoothed estimate-can be approximated as:

9 $$\:\begin{array}{c}{N}_{\text{e}\text{f}\text{f}}=\frac{1}{1-{\lambda\:}_{t}}\end{array}$$

This standard approximation provides a quantitative basis for setting the boundaries of $\:{\lambda\:}_{t}$. Detailed experimental validation of this range is presented in the Experimental Results and Analysis section.

Lower Bound (0.1):

When the smoothing factor $\:{\lambda\:}_{t}<0.1$, the effective memory length becomes $\:{N}_{\text{e}\text{f}\text{f}}\approx\:10$ steps or fewer. This approaches an under-smoothing regime ($\:{\lambda\:}_{t}\approx\:0$), where historical gradient information is almost entirely discarded ($\:{b}_{t}\approx\:{g}_{t}$). Such insufficient memory makes parameter updates highly susceptible to stochastic gradient noise, causing instability and even divergence during training. The lower bound of 0.1 ensures a minimum effective memory of approximately 10 steps, providing sufficient inertia to suppress high-frequency noise in stochastic optimization, particularly in flat or noisy regions of the loss surface.

Upper Bound (0.9):

When the smoothing factor $\:{\lambda\:}_{t}>0.9$, the effective memory length becomes $\:{N}_{\text{e}\text{f}\text{f}}\approx\:10$ steps or more. This approaches an over-smoothing regime ($\:{\lambda\:}_{t}\approx\:1$), where the smoothed gradient $\:{b}_{t}$ is overly dependent on historical states ($\:{b}_{t}\approx\:{b}_{t-1}$), introducing significant lag (inertia) and severely delaying the optimizer’s response to abrupt gradient changes. Such delays are particularly detrimental in non-stationary phases or near sharp minima. The upper bound of 0.9 sets the maximum effective memory to about 10 steps, preventing excessive inertia and ensuring that the optimizer remains agile in tracking gradient dynamics.

The smoothed gradient is then updated using:

10 $$\:\begin{array}{c}{b}_{t}={\lambda\:}_{t}{b}_{t-1}+\left(1-{\lambda\:}_{t}\right){g}_{t}\end{array}$$

where $\:{b}_{t}$ is the momentum-smoothed gradient incorporating the Semi-adaptive mechanism.

To further improve the stability of gradient updates, the smoothed gradient is normalized so that its distribution remains within a stable range:

11 $$\:\begin{array}{c}\stackrel{\sim}{{g}_{t}}=\frac{{b}_{t}}{\sigma\:\left({b}_{t}\right)+\epsilon\:}\end{array}$$

where $\:\sigma\:\left({b}_{t}\right)$ denotes the standard deviation of the smoothed gradient. This normalization ensures that parameter updates remain consistent in magnitude across different layers or scales.

Furthermore, dynamic gradient scaling is employed to regulate the update step size:

When the gradient variance is too large, the scaling factor is limited to a minimum value to prevent instability from overly aggressive updates.

When the gradient variance is too small, the scaling factor is limited to a maximum value to ensure sufficient update magnitude and avoid premature convergence to local optima.

This mechanism enables the optimizer to maintain consistent behavior across a wide range of gradient distributions, thus enhancing both optimization stability and generalization performance. The dynamic gradient scaling formula is as follows:

12 $$\:\begin{array}{c}scaled\_grad=improved\_grad\times\:clip(\frac{1}{grad\_std},{min},{max})\end{array}$$

The formula $$\:grad\_std=\sigma\left({b}_{t}\right)+\varepsilon\:$$.

Finally, the Sigmoid activation function is utilized to balance between noise suppression and adaptation speed, allowing for smooth transitions in the smoothing strength during training. This design allows the model to avoid oscillations or divergence in early stages while accelerating convergence in later stages.

The proposed semi-adaptive gradient smoothing mechanism plays a crucial role in mitigating oscillatory dispersion within regions of pathological curvature, a common challenge in non-convex optimization landscapes characterized by extremely high Hessian condition numbers^31,32^. This mechanism operates by dynamically adjusting the effective momentum buffer size based on the instantaneous gradient magnitude ($\:{g}_{t}$), thereby modulating the balance between rapid response and noise suppression in a unified manner. Specifically, when the optimizer encounters steep directions—often corresponding to narrow ravines with large eigenvalues LLL of the Hessian—the smoothing factor $\:{\lambda\:}_{t}$​ rapidly approaches its lower bound (≈ 0.1) according to Eq. (7) and Eq. (8). This adjustment reduces the weight of the historical smoothed gradient $\:{b}_{t-1}$​ in Eq. (10), effectively shrinking the momentum buffer and allowing the update $\:{b}_{t}$​ to respond quickly to the current gradient $\:{g}_{t}$​. Such responsiveness alleviates the conflict between strong, up-to-date directional signals and potentially outdated or misaligned momentum, thus reducing overshooting and oscillatory behaviour along the steep walls of pathological ravines. Conversely, in flatter regions—typically associated with small Hessian eigenvalues $\:\mu\:$—the gradient magnitude is modest, and $\:{\lambda\:}_{t}$​ naturally approaches its upper bound (≈ 0.9), enlarging the momentum buffer and producing a heavily smoothed estimate of recent gradients. This smoothing effectively filters out high-frequency stochastic noise inherent in mini-batch SGD, providing a more stable update direction and preventing jitter in low-curvature regions. The constraint $\:{\lambda\:}_{t}\in\:\left[\text{0.1,0.9}\right]$ is essential for ensuring robustness: the lower bound avoids under-smoothing in noisy steep gradients, while the upper bound avoids over-smoothing that might delay necessary directional corrections.

From a theoretical perspective, the stabilizing effect of this mechanism can be analyzed using a simplified pathological curvature model where the local loss surface is approximated by a quadratic form $\:f\left(x\right)=\frac{1}{2}{x}^{\text{T}}Hx$,with H positive-definite but ill-conditioned (0 < $\:\mu\:$ ≪ L). In the steepest direction with curvature L, the momentum update with smoothing $\:{\lambda\:}_{t}$ follows Eq. (10), $\:{g}_{t}=L\left({x}_{t}\text{}-x\text{*}\right)$. Substituting into $\:\:{x}_{t+1}={x}_{t}-{\upeta\:}{b}_{t}\:$ yields a second-order linear difference equation whose stability requires $\:\left|1-{\upeta\:}L+{\uplambda\:}\right|<1$^33^. For large LLL, a smaller $\:{\lambda\:}_{t}$ reduces the effective amplification term, shrinking the spectral radius and thus suppressing oscillations. In the flat direction with curvature $\:\mu\:$, a larger $\:{\lambda\:}_{t}$ increases the averaging effect and reduces stochastic variance, approximately $\:\text{Var}\left({b}_{t}\right)\:\approx\:\:\frac{{\left(1-{\lambda\:}_{t}\right)}^{2}}{1-{{\lambda\:}_{t}}^{2}}{{\upsigma\:}}_{g}^{2}$. By dynamically adjusting $\:{\lambda\:}_{t}$ based on $\:\left|{g}_{t}\right|$ the spectral radius in steep directions is minimized while variance in flat regions is suppressed, thereby ensuring both stability and noise robustness. This theoretical derivation directly validates the mechanism’s ability to mitigate oscillatory dispersion in pathological curvature regions without sacrificing convergence efficiency.

In summary, the semi-adaptive gradient smoothing controller adaptively modulates the influence of historical gradients in response to real-time curvature conditions, enabling it to balance rapid adaptation to strong directional cues with suppression of stochastic noise, and thus effectively stabilizing the optimization trajectory in highly ill-conditioned regions of deep neural network training.

## Adaptive variance correction mechanisms

The gradient stabilization and dynamic rectification mechanism is designed to enhance early-stage training stability by adjusting the effective learning rate in response to bias in moment estimation. In conventional Adam, the use of a fixed learning rate or simple decay schedule often leads to mismatches between gradient magnitudes and step sizes, particularly during the early iterations when the second-order moment estimate remains biased^1,2^.

To address this issue, a bias correction strategy is employed, where the first-order moment estimate is adjusted using a time-dependent correction factor. This correction compensates for the initial underestimation caused by exponential moving averages initialized at zero. The correction formula is given as follows:

13 $$\:\begin{array}{c}\stackrel{\wedge\:}{{m}_{t}}=\frac{{m}_{t}}{1-{\beta\:}_{1}^{t}}\end{array}$$

By applying this correction, the influence of early momentum bias is reduced, resulting in more stable parameter updates during the initial optimization steps.

Although the existing correction factors alleviate the variance problem in the initial training stage, they fail to dynamically adjust the correction strategy based on gradient variation. To address this, a gradient-stabilized dynamic learning rate rectification mechanism is introduced in this work. The mechanism retains the original bias correction strategy and further incorporates a dynamic variance sensing module to adjust the learning rate adaptively based on the training step and the variance of the gradients.

A key component of this mechanism is the correction factor $\:{\rho\:}_{t}$, defined as:

14 $$\:\begin{array}{c}{\rho\:}_{{\infty\:}}=\frac{2}{1-{\beta\:}_{2}}-1\end{array}$$

15 $$\:\begin{array}{c}{\rho\:}_{t}={\rho\:}_{{\infty\:}}-\frac{2t{\beta\:}_{2}^{t}}{1-{\beta\:}_{2}^{t}}\end{array}$$

Definition of Variables:

### $\:{\rho\:}_{t}$:

the dynamic correction factor at training step, derived from the second-order moment estimator.

### $\:{\rho\:}_{{\infty\:}}$:

the theoretical upper bound of $\:{\rho\:}_{t}$ as $t \rightarrow\infty$ .

### $\:{\beta\:}_{2}$:

the exponential decay rate for the second-order moment, typically set to 0.999.

### $\:{\beta\:}_{1}$:

the exponential decay rate for the first-order moment.

*t*: the current training step.

### $\:adaptive\_lr$:

the adjusted learning rate based on $\:{\rho\:}_{t}$.

### $\:\text{group}\left[{\text{'lr}}^{{\prime\:}}\right]$:

the base learning rate from the optimizer configuration.

### $\:step\_size$

the effective step size after correction.

### $\stackrel{\wedge\:}{{v}_{t}}$:

the bias-corrected second-order moment estimate (variance).

### $\stackrel{\wedge\:}{{g}_{t}}$:

the normalized and smoothed gradient.

### $\varepsilon$:

a small constant for numerical stability (e.g.,10 − 8).

### $\:{\theta}_{t}$

the updated model parameters at step.

### $\:{\theta}_{t-1}$

the model parameters from the previous step.

Under a standard configuration of $\:{\beta\:}_{2}$ = 0.999, $\:{\rho\:}_{{\infty\:}}$ ≈ 1999, and it can be analytically shown that when $\:t\in\:\left[\text{50,70}\right]$, $\:{\rho\:}_{t}\approx\:5$. This value represents a natural transitional phase, where the early-stage bias in the second-order moment estimation becomes sufficiently small, and the optimization enters a more stable update region. Thus, the threshold $\:{\rho\:}_{t}>5$ was not arbitrarily chosen, but is theoretically grounded as it effectively separates the bias-dominated phase from the later stable training phase, enabling an adaptive and efficient $\:step\_size$ switching strategy.

During early training, when $\:{\rho\:}_{t}>5$, an adaptive learning rate adaptive_lr is computed and used to adjust the step size:

16 $$\:\begin{array}{c}\:adaptive\_lr=\sqrt{\frac{\left({\rho\:}_{t}-4\right)\left({\rho\:}_{t}-2\right){\rho\:}_{{\infty\:}}}{\left({\rho\:}_{{\infty\:}}-4\right)\left({\rho\:}_{{\infty\:}}-2\right){\rho\:}_{t}}}\end{array}$$

This value is combined with the first-order bias correction factor $\:1-{\beta\:}_{1}^{t}$ to produce a dynamic update step:

17 $$\:\begin{array}{c}step\_size=\frac{group{[}^{{\prime\:}}l{r}^{{\prime\:}}]\cdot\:\:adaptive\_lr}{1-{\beta\:}_{1}^{t}}\end{array}$$

The final parameter update is then performed as:

18 $$\:\begin{array}{c}denom=\sqrt{\stackrel{\wedge\:}{{v}_{t}}}+\varepsilon\:\end{array}$$

19 $$\:\begin{array}{c}{\theta\:}_{t}={\theta\:}_{t-1}-\frac{step\_size}{denom}\cdot\:\stackrel{\wedge\:}{{g}_{t}}\end{array}$$

When $\:{\rho\:}_{t}\le\:5$, the optimizer adopts a simpler fixed-step update:

20 $$\:\begin{array}{c}{\theta\:}_{t}={\theta\:}_{t-1}-group{[}^{{\prime\:}}l{r}^{{\prime\:}}]\cdot\:\stackrel{\wedge\:}{{g}_{t}}\end{array}$$

To achieve a balance between fast convergence and fine-grained optimization, this paper introduces a staged optimization strategy. In the early training phase, a relatively aggressive learning rate is adopted to accelerate convergence and help the model escape suboptimal regions. As training progresses, the algorithm transitions to a more adaptive learning stage by coupling bias correction with gradient-sensitive learning rate adjustment. This dynamic step-size modulation helps to refine model parameters near the optimum and mitigates the risk of oscillations caused by overly large updates. Experimental results show that this two-phase optimization scheme not only ensures rapid convergence at the initial stage but also significantly enhances convergence stability and final accuracy in the later stage, outperforming traditional fixed-step optimizers in both speed and precision.

## The BDS-Adam algorithm

The traditional Adam optimization algorithm, a widely used adaptive method in deep learning, is recognized for its ability to dynamically adjust learning rates and accelerate early-stage convergence^1^. However, several critical limitations persist in practical applications. One major issue is that Adam’s parameter update step size is not guaranteed to decrease monotonically, which can hinder stable convergence toward a global optimum^8^.

Additionally, Adam^34^ is prone to oscillations in parameter updates, especially when hyperparameters such as the learning rate or momentum factors are improperly configured. These oscillations result from the interaction between the adaptive learning rate mechanism and noisy gradients—particularly when the estimates of the second-order moments are inaccurate or unstable^3,6^.

Compared to stochastic gradient descent (SGD), models trained with Adam often show inferior generalization performance on unseen test data. This is not because gradients are needed at test time, but because Adam’s training dynamics tend to favor sharp minima in the loss surface. These sharp regions, which are heavily influenced by the optimizer’s reliance on historical gradient statistics (e.g., exponential moving averages of squared gradients), exhibit high curvature and are more sensitive to small perturbations, leading to degraded robustness under distribution shifts^28^. In contrast, SGD—with its consistent update size and stochastic noise—tends to converge to flatter minima, which are associated with better generalization.

Furthermore, Adam exhibits higher sensitivity to weight initialization than SGD or other momentum-based optimizers. Variations in initial parameter values can lead to significantly different learning trajectories and final model performance, particularly in high-dimensional and non-convex settings^35^.

For large-scale models and complex datasets, Adam’s adaptive learning rate mechanism can become unstable. The per-parameter variance estimates may amplify low-frequency gradient noise, causing unintended fluctuations in the effective learning rates. In high-dimensional spaces, such instability becomes compounded across parameters and layers, resulting in oscillatory update patterns that negatively impact convergence^36^.

To address these challenges, an improved variant named BDS-Adam is proposed. This algorithm integrates several novel components, including Semi-Adaptive gradient smoothing, gradient normalization, dynamic gradient scaling, and a gradient stabilization and rectification mechanism.

**Fig. 1:**
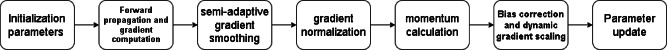
Flowchart of BDS-Adam algorithm.

The complete framework is illustrated in Fig. 1.

Initialization: When initializing the BDS-Adam optimizer, a set of hyperparameters must be specified, including learning rate (lr), momentum coefficients (betas), numerical stability constant (eps), weight decay coefficient (weight_decay), semi-adaptive smoothing base factor, minimum and maximum gradient scaling factors (min_gradient_scale, max_gradient_scale). Input validation is performed to ensure all values are within acceptable ranges. If a parameter’s internal state is uninitialized, it is set to zero.

Gradient Computation: The gradient grad is obtained via backpropagation. If the gradient is a sparse tensor, an error is raised, as the algorithm does not support sparse gradients. If weight decay is enabled, the decay term is added to the gradient.

semi-adaptive gradient smoothing: The smoothed gradient is computed using a dynamic smoothing factor. First, the L2 norm of the current gradient is calculated. Then, the smoothing factor $\:{\lambda\:}_{t}$ is generated using a tanh-based transformation of the gradient norm. When the gradient is large (typically in early training), $\:{\lambda\:}_{t}$ is reduced to retain more instantaneous information; when the gradient is small (later training), $\:{\lambda\:}_{t}$ increases to suppress noise. The smoothing factor is constrained within [0.1, 0.9] to ensure stability.

Gradient Normalization: The smoothed gradient is normalized by its standard deviation (plus a small constant, 1e-8) to obtain a dimensionally consistent update direction. This ensures different parameters have balanced update magnitudes.

Momentum Estimation: First-order (mean) and second-order (variance) moment estimates are updated using exponential moving averages controlled by $\:{\beta\:}_{1}$ and $\:{\beta\:}_{2}$, respectively.

Bias Correction and Dynamic Gradient Scaling: The bias in the first-order moment estimate is corrected using the factor ($\:1-{\beta\:}_{1}^{t}$). The smoothed gradient is also scaled based on its standard deviation, with the scaling factor clipped to a pre-specified range [min_gradient_scale, max_gradient_scale] to avoid overly small or large updates.

Variance Rectification and Parameter Update: To ensure stable updates across training stages, BDS-Adam employs a two-phase update strategy based on the dynamic threshold ρₜ. As previously established, ρₜ reflects the reliability of the variance estimate. When ρₜ ≤ 5, the optimizer uses a simplified SGD-like update with the scaled gradient to enhance early-stage stability. Once ρₜ exceeds 5, a full bias-corrected and variance-scaled update is applied using the normalized gradient. This conditional strategy balances fast convergence at the beginning with precise optimization near the minima.

The specific algorithm is shown in Algorithm 1.

**Figure:**
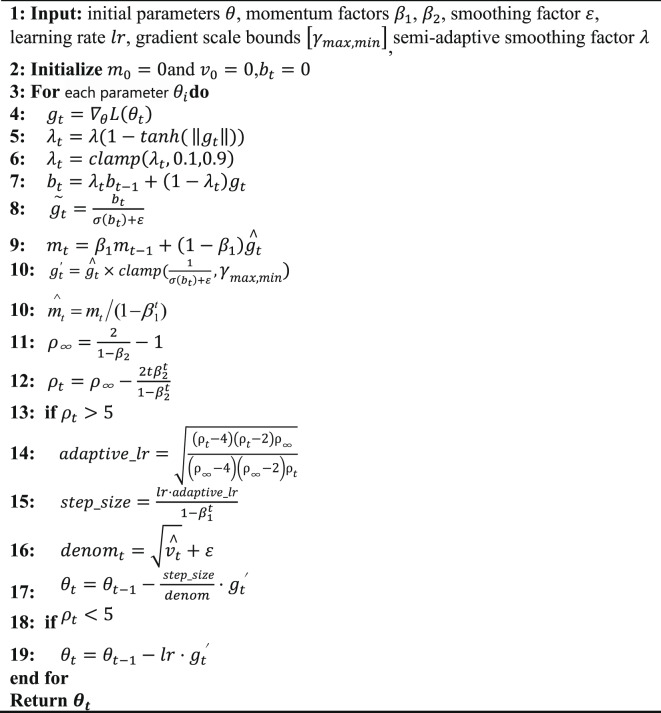
**Algorithm 1:** (BDS-Adam)

### Experimental design and analysis of results

#### Configuration of experimental environment

All experiments were implemented in Python 3.10 using the PyTorch 2.0.1 deep learning framework. The proposed BDS-Adam optimizer, which integrates a semi-adaptive gradient smoothing module and a gradient stabilization and dynamic rectification mechanism, was applied throughout the evaluation. The software environment was managed using the PyTorch Lightning 2.1.2 framework, and computations were accelerated by CUDA version cu118. Detailed software and hardware configurations are listed in Table 1.

**Table 1:** Experimental software and hardware configuration.

Software and Hardware	Version
Python	3.10
Torch	2.0.1
Torchvision	0.15.0
Lightning	2.1.2
Cuda	cu118

To comprehensively evaluate the generalization performance of BDS-Adam under different data complexities, classification tasks were performed on three image datasets: MNIST, CIFAR-10, and Stomach.

- MNIST is a widely used benchmark dataset for handwritten digit recognition. It contains 70,000 grayscale images (28 × 28 pixels) representing digits from 0 to 9 in diverse handwriting styles.
- CIFAR-10 consists of 60,000 (32 × 32 pixels) color images across 10 object categories, covering natural scenes with higher semantic variability.
- Stomach is a medical imaging dataset consisting of 1,885 endoscopic images representing eight categories of digestive tract lesions (e.g., hemorrhoids, polyps, ulcers). All images were resized to 224 × 224 pixels during preprocessing to ensure consistency.

The three datasets differ significantly in image resolution, domain complexity, class diversity, and data volume. These variations are summarized in Table 2. MNIST and CIFAR-10 serve as standard benchmarks, while Stomach represents a real-world low-resource medical scenario. This diverse evaluation setup enables a thorough examination of the optimizer’s performance across low-level (grayscale digits), medium-level (natural color scenes), and high-level (specialized medical) data modalities.

**Table 2:** Dataset characteristics summary.

Data set	Number of samples	Training set	Test set	Validation set	Category	Data characteristics
MNIST	70,000	55,000	5000	10,000	10	Image size unification, data diversity, moderate data volume
CIFAR10	60,000	45,000	5000	10,000	10	RGB image, relatively small scale, small image size
Stomach	1885	900	485	500	8	RGB image, few categories, recognition difficulty

## Experimental results and analysis

To comprehensively evaluate the performance of the proposed BDS-Adam optimizer, a multi-dimensional experimental framework was constructed. Classical optimization algorithms including SGD, Adagrad, Adam, AdamW, and NAdam were selected as baselines for comparison. Under strictly controlled initial conditions, these optimizers were evaluated across three key dimensions: convergence speed (i.e., loss descent trajectory), generalization ability (i.e., test set accuracy), and training stability (i.e., loss variance).

Experiments were conducted on three datasets of increasing complexity—MNIST, CIFAR-10, and a gastric pathology image dataset (Stomach)—under both optimal and suboptimal learning rate configurations (0.001 and 0.0001). This was designed to analyze the performance robustness of the optimizers across different gradient dynamics and hyperparameter sensitivity scenarios.

To ensure fair and reproducible evaluation, a standardized experimental protocol was adopted:

- Optimizers: The experiments involved ten optimizers—SGD, Adagrad, Adam, AdamW, NAdam, RAdam, AdaBelief, LAMB, Lookahead, and the proposed BDS-Adam—for comparative analysis. For RAdam, AdaBelief, LAMB, and Lookahead, experiments were conducted for 50 epochs, as convergence was observed within this range across all datasets.
- Epochs: The number of training epochs was fixed at 100 for the baseline comparison experiments and set to 50 for additional optimizer evaluation, ensuring both thoroughness and computational efficiency.
- Batch size: The batch size was uniformly set to 128 to ensure consistent mini-batch processing across optimizers.
- Learning rate: An initial learning rate of 0.001 was adopted for all optimizers in the main comparison. To evaluate robustness under suboptimal hyperparameter settings, additional experiments were conducted at a lower learning rate of 0.0001.
- Parameter tuning: For BDS-Adam, grid search was performed to determine optimal configurations of the base smoothing factor (i.e., semi-adaptive smoothing coefficient$\:{\lambda\:}_{t}$) and dynamic gradient scaling bounds. Experiments were conducted with fixed smoothing coefficients $\:{\lambda\:}_{t}\in\:\text{0.1,0.5,0.9}$, as well as with a dynamic adaptive range $\:{\lambda\:}_{t}\in\:\left[\text{0.1,0.9}\right]$, to validate the theoretical constraint range and its empirical performance.
- Evaluation metrics: A multi-objective evaluation scheme was used across datasets, incorporating classification accuracy (Acc), training loss (Loss), and convergence stability. Results demonstrate that BDS-Adam consistently outperforms baseline optimizers in suppressing biased gradient estimations and enhancing generalization.

To ensure fair and reproducible evaluation of the BDS-Adam optimizer across datasets of varying complexity, we adopted appropriate and well-established neural network architectures for each dataset:

- MNIST: We used a custom-designed lightweight convolutional network named SimpleDenseNet, consisting of two convolutional layers (with 32 and 64 filters, kernel size 3 × 3, followed by ReLU activation), a 2 × 2 max-pooling layer, a dropout layer (rate = 0.25), a dense block with fully connected layers (128 and 64 units respectively), and a final softmax output layer for 10-class classification. This architecture is designed to balance model capacity and training efficiency on low-resolution digit data.
- CIFAR10: We employed the MobileNetV2 architecture, a lightweight yet powerful convolutional neural network optimized for image classification tasks on resource-constrained devices. The network incorporates depthwise separable convolutions and inverted residuals with linear bottlenecks. It was trained from scratch without ImageNet pretraining to fairly assess the impact of different optimizers.
- Stomach Dataset: For the medical image classification task, we also used MobileNetV2, but with ImageNet-pretrained weights to enhance performance given the limited dataset size. The final fully connected classification layer was replaced to match the 8-class gastric pathology categories. Data augmentation techniques such as random horizontal flipping, random cropping, and color jittering were applied to enhance generalization.

All models were implemented in PyTorch. For each dataset, the same network was used across all optimizer configurations to ensure that performance differences can be attributed solely to the optimization strategy. To ensure computational fairness, all optimizers were trained under identical hardware settings, with matched batch sizes and model configurations.

**Table 3:** Comparison of experimental results of different algorithms on MNIST.

Data set	Optimization Algorithm	Accuracy	Loss
MNIST	SGD	97.55%	0.077
	Adagrad	98.00%	0.068
	Adamw	98.56%	0.063
	Adam	98.61%	0.061
	Nadam	98.59%	0.057
	BDSAdam	98.69%	0.064

**Fig. 2:**
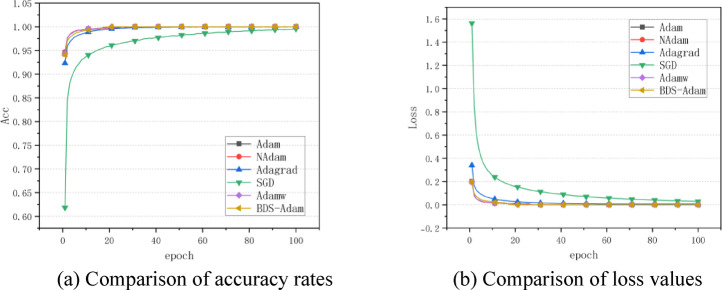
Performance comparison of various algorithms on MNIST dataset. (**a**) Comparison of accuracy rates (**b**) Comparison of loss values.

According to the optimized algorithm comparison experimental results shown in Fig. 2(a), (b), the BDS-Adam algorithm demonstrates multi-dimensional performance advantages in the MNIST handwritten digit classification task. Observed from the accuracy curve (Fig. 2a), BDS-Adam shows stable convergence characteristics over 100 training cycles, and its final test accuracy reaches 98.69% (Table 3). As shown in the loss evolution plot (Fig. 2b), most optimizers, including BDS-Adam and Adam, did not exhibit significant oscillations during training. However, BDS-Adam achieved a smoother convergence trajectory with consistently lower loss values, indicating enhanced training stability.

As the experimental data in Table 4, the BDS-Adam accuracy value is 87.31% on the dataset of CIFAR10, which is 9.27% higher than the Adam accuracy. The BDS-Adam loss value is 0.756, which is 0.184 lower than the Adam loss value.

**Table 4:** Comparison of experimental results of different algorithms on CIFAR10.

Data set	Optimization Algorithm	Accuracy	Loss
CIFAR10	SGD	41.23%	1.639
	Adagrad	50.05%	1.394
	Adamw	78.84%	0.889
	Adam	78.04%	0.940
	Nadam	57.65%	1.331
	BDSAdam	87.31%	0.756

**Fig. 3:**
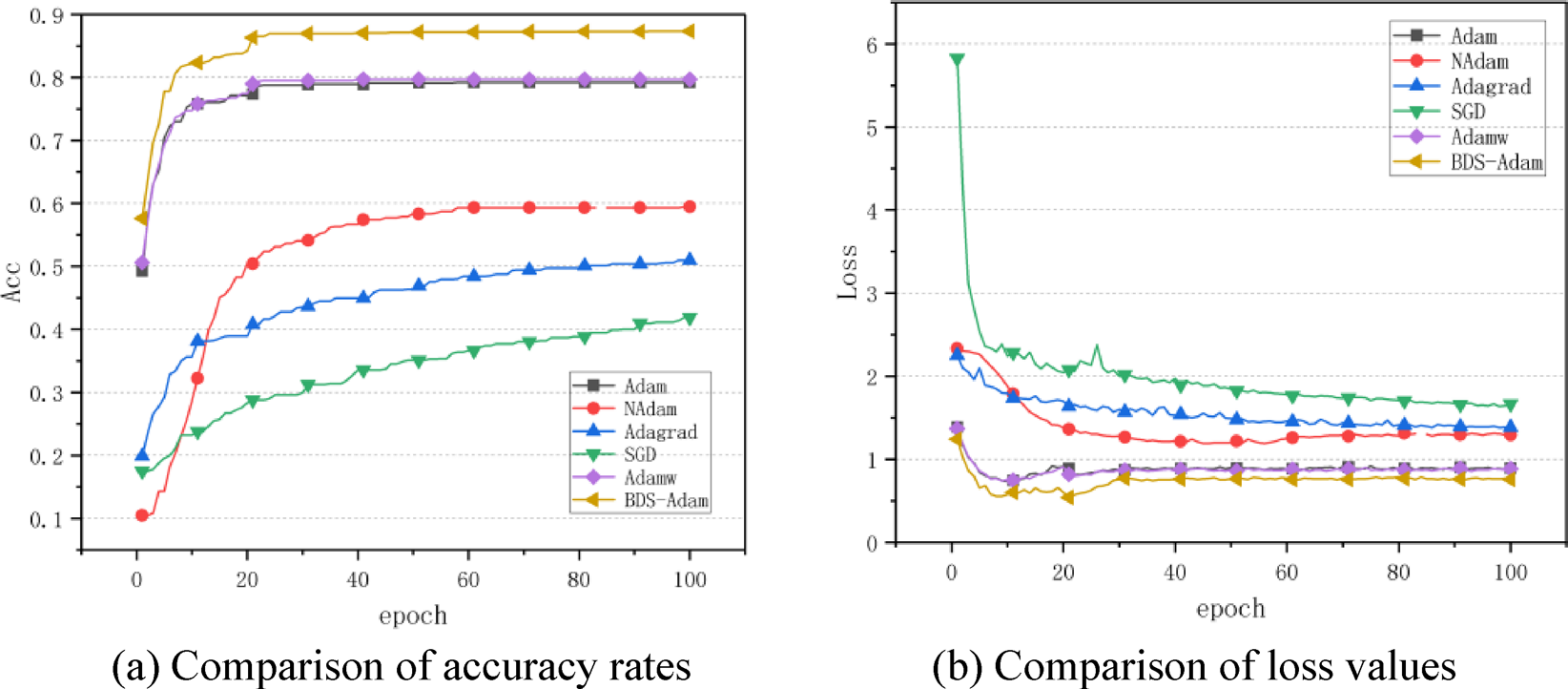
Performance comparison of various algorithms on CIFAR10 dataset. (**a**) Comparison of accuracy rates, (**b**) Comparison of loss values.

According to the results of the optimized algorithm comparison experiments on the CIFAR-10 dataset in Fig. 3, BDS-Adam shows significant performance improvement in complex image classification tasks. As seen from the accuracy evolution curve, BDS-Adam shows a continuous upward trend within 100 training cycles, which is a significant improvement over the benchmark Adam algorithm. The loss function comparison further shows that the cross-entropy loss convergence of BDS-Adam is significantly lower than that of Adam, and the stability of the convergence process is significantly better than that of the other compared algorithms.

To further evaluate the competitiveness of BDS-Adam, we compare it with several recent Adam-based optimizers, including LAMB, Lookahead, RAdam, and AdaBelief, on the CIFAR-10 dataset. These methods have been widely adopted in various deep learning tasks for their respective enhancements over standard Adam, such as layer-wise adaptation (LAMB), long-term stability (Lookahead), rectified variance (RAdam), and belief-based update (AdaBelief).

**Fig. 4:**
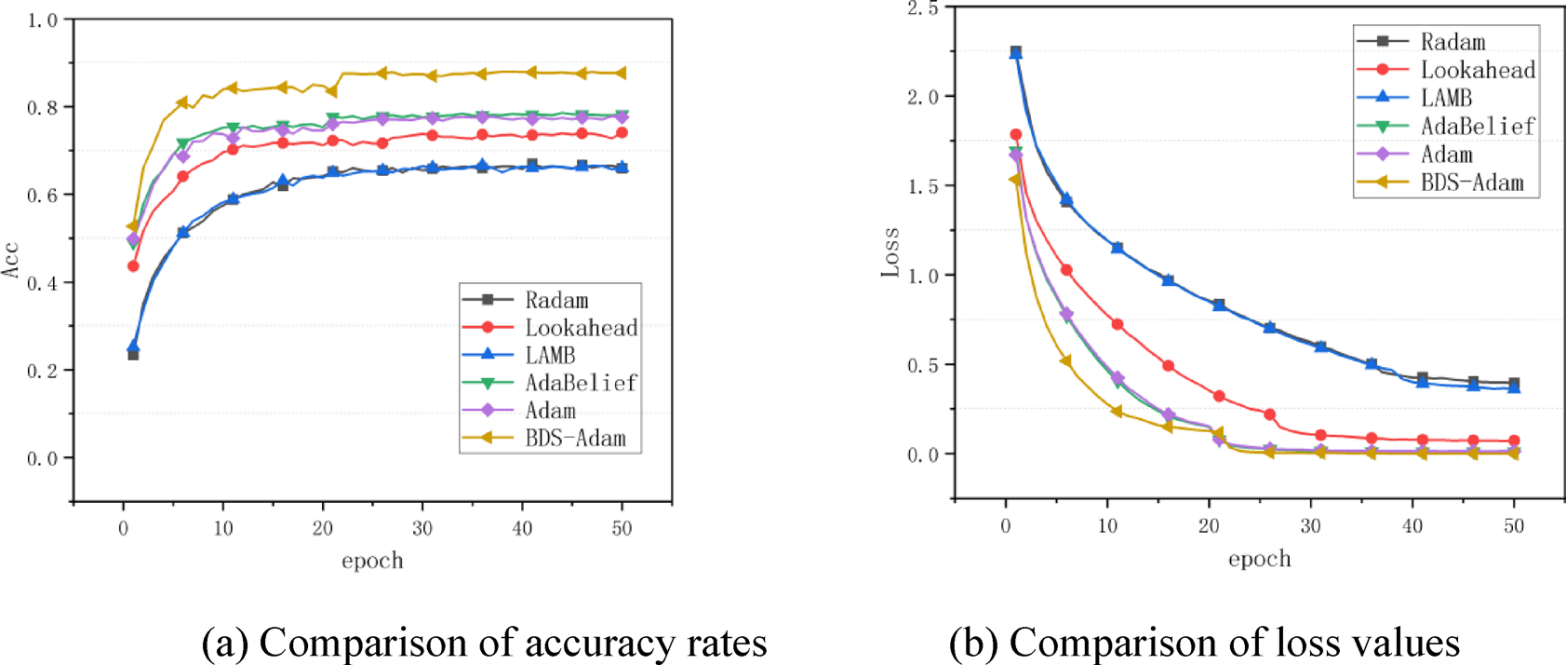
Performance comparison of recent Adam variants on CIFAR10 dataset. (**a**) Comparison of accuracy rates, (**b**) Comparison of loss values.

The comparison results are illustrated in Fig. 4, which presents both the test accuracy and test loss curves across 50 training epochs under the same learning rate and training settings.

As shown in the accuracy curve (Fig. 4a), BDS-Adam achieves the highest test accuracy and demonstrates faster convergence than all compared variants. Unlike RAdam and LAMB, which show slower initial growth, or Lookahead, BDS-Adam maintains a stable increase in performance throughout the training process.

In the loss comparison (Fig. 4b), BDS-Adam reaches a lower cross-entropy loss much earlier, indicating faster convergence. AdaBelief and Adam, although effective in some stages, still converges to a higher final loss. These results confirm that BDS-Adam not only surpasses the baseline Adam optimizer, but also consistently outperforms several of its advanced variants in both convergence speed and generalization ability.

- Robustness under Suboptimal Hyperparameter Settings:

To evaluate the robustness of different optimizers in non-ideal training conditions, we conducted experiments using a suboptimal learning rate of 0.0001, which deviates from the typical best-tuned values. Table 5 reports the final test accuracy and cross-entropy loss on the CIFAR-10 dataset under this setting.

**Table 5:** Performance of different optimizers under suboptimal learning rate (0.0001) on CIFAR-10.

Data set	Optimization Algorithm	Accuracy	Loss
CIFAR10	Lookahead	72.76%	1.032
	LAMB	65.85%	1.088
	AdaBelief	78.64%	0.914
	Adam	78.04%	0.943
	Radam	67.42%	1.026
	BDSAdam	86.71%	0.854

As shown in Table 5, while most optimizers suffer noticeable performance drops under suboptimal settings, BDS-Adam maintains significantly higher test accuracy (86.71%) and lower loss (0.854). This indicates that BDS-Adam is more robust to learning rate misconfigurations, thanks to its dual adaptive mechanisms—namely, the semi-adaptive momentum smoothing controller and gradient-aware scaling, which dynamically stabilize updates even when the hyperparameter tuning is not ideal.

- Ablation Study on CIFAR-10:

To assess the individual contributions of the proposed components in BDS-Adam, we conducted ablation experiments on the CIFAR-10 dataset. Although our main experimental results are based on the Stomach dataset, we chose CIFAR-10 for the ablation study due to its moderate task complexity, faster training cycles, and higher reproducibility. These characteristics make it particularly suitable for analyzing optimizer behavior and isolating the effects of individual algorithmic components. The insights derived from CIFAR-10 remain representative and valid for understanding the internal mechanisms of BDS-Adam.

In this study, we selectively removed the following key modules:

1. Adaptive Momentum Smoothing Controller: disables the semi-adaptive-style smoothing mechanism;
2. Gradient Normalization: removes the standard deviation normalization applied to smoothed gradients;
3. Dynamic Gradient Scaling: removes the gradient scaling factor that adjusts updates based on gradient statistics;
4. Adaptive Parameter Update Rule: disables the RAdam-style adaptive update mechanism based on local curvature.

**Fig. 5:**
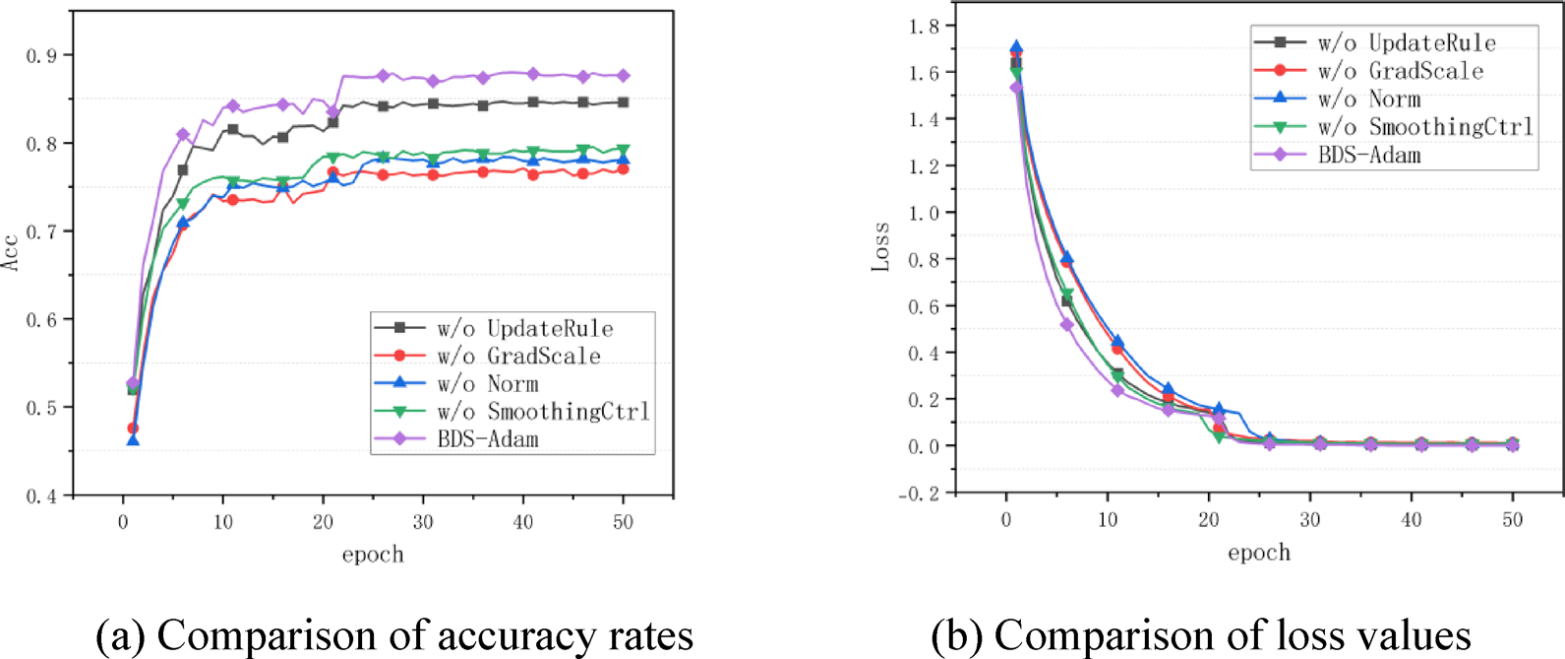
Ablation Study on CIFAR-10 dataset. (**a**) Comparison of accuracy rates, (**b**) Comparison of loss values.

The experimental results are shown in Fig. 5, which plot the evolution of classification accuracy and cross-entropy loss over 50 training epochs.

As shown in Fig. 5, the complete version of BDS-Adam outperforms all ablated variants in both accuracy and convergence. The removal of the adaptive momentum smoothing controller and parameter update rule results in the largest drop in final accuracy and slower convergence. This indicates that these modules are most critical to the effectiveness of the optimizer. Meanwhile, gradient normalization and dynamic scaling also contribute positively by stabilizing early-stage training and improving generalization.

**Fig. 6:**
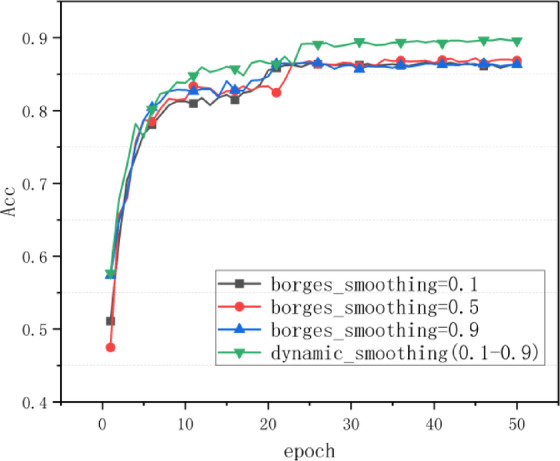
Test accuracy of BDS-Adam with fixed vs. dynamic smoothing coefficients on CIFAR-10.

To validate the rationality of the dynamic smoothing coefficient range [0.1, 0.9] in the semi-adaptive smoothing mechanism, we conducted comparative experiments. The smoothing coefficient was set to fixed values of 0.1, 0.5, and 0.9, as well as dynamically adjusted within the interval [0.1, 0.9]. As shown in Fig. 6, the BDS-Adam optimizer with dynamic coefficient adjustment achieved the highest test accuracy, outperforming all fixed coefficient settings.

This result demonstrates that constraining the smoothing coefficient within the [0.1, 0.9] interval allows effective balance between gradient update stability and convergence speed, providing empirical support for the theoretically proposed parameter range.

**Fig. 7:**
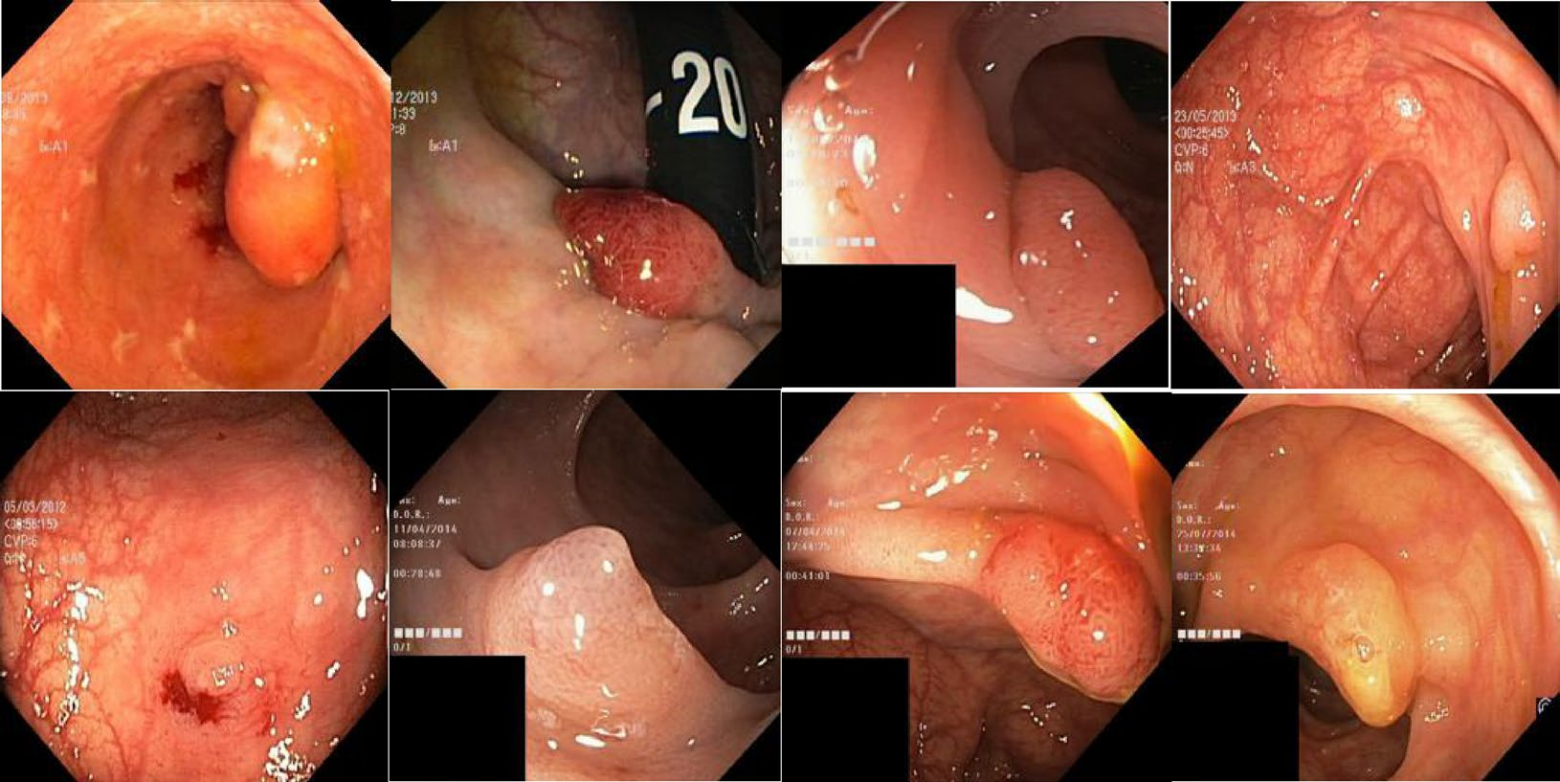
Image of the Stomach dataset.

In the field of medical image analysis, automated detection techniques for gastric lesions are of great clinical significance, but still face challenges such as the scarcity of high-quality labeled data and the difficulty of extracting complex pathological features. The Stomach dataset used in this study is illustrated in Fig. 7, which contains 3,200 high-resolution gastric endoscopic images covering four types of pathological features, including normal tissue, early gastric cancer, ulcers, and polyps.

As shown in the experimental data in Table 6, BDS-Adam achieves an accuracy of 79.4% on the Stomach dataset, which is 3.0% higher than that of Adam. The loss value of BDS-Adam is 0.591, which is 0.193 lower than that of Adam. BDS-Adam achieves the optimal accuracy on all the three datasets, demonstrating its excellent performance in processing different types of datasets (including basic black-and-white image data and more complex color RGB image data), verifying its outstanding generalization ability.

**Table 6:** Comparison of experimental results of different algorithms on Stomach.

Data set	Optimization Algorithm	Accuracy	Loss
Stomach	SGD	72.60%	0.894
	Adagrad	69.80%	0.921
	Adamw	76.20%	0.778
	Adam	76.40%	0.784
	Nadam	76.80%	0.789
	BDSAdam	79.40%	0.591

**Fig. 8:**
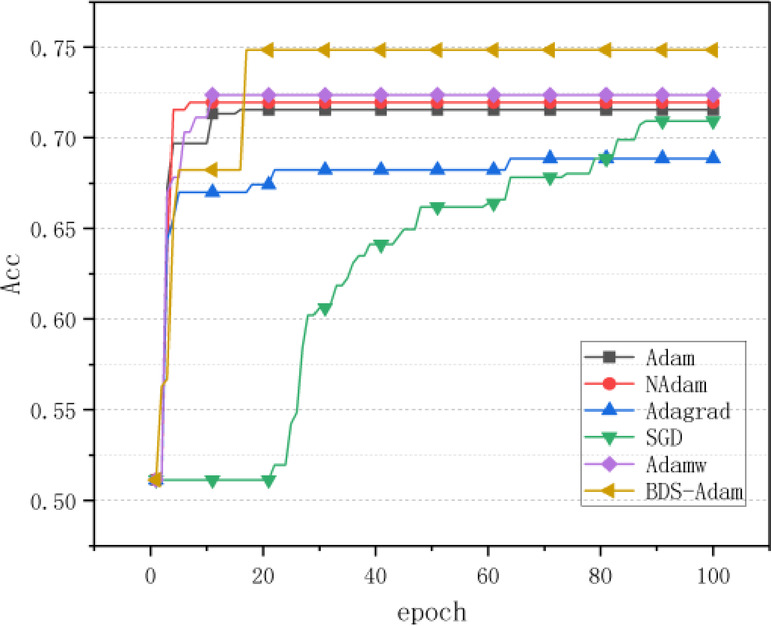
Comparison of accuracy of various algorithms on Stomach dataset.

As shown in Fig. 8, in the experimental results on the Stomach dataset, the classification accuracy of SGD and Adagrad significantly lags behind other optimizers, with an absolute performance gap exceeding 15% compared to the optimal method.

Notably, the BDS-Adam algorithm exhibits breakthrough convergence characteristics during the early stage of training (10–20 epochs), achieving iterative accuracy improvements of 3.2% and 4.1% over the suboptimal algorithms NAdam and AdamW, respectively. This indicates its effectiveness in overcoming the gradient disorientation problem observed in traditional Adam-based improvement algorithms during early training. The advantage originates from its dynamic gradient adjustment architecture: through the semi-adaptive gradient smoothing mechanism, the hyperbolic tangent function dynamically calibrates the historical gradient retention ratio (within the interval [0.1, 0.9]), enabling nonlinear smoothing intensity adjustment by real-time sensing of gradient patterns, which significantly enhances robustness against noise. Simultaneously, the gradient normalization technique, driven by standard deviation–based scale invariance, constrains the gradient distribution and establishes a stable feature learning space. This dual regulation mechanism substantially improves feature exploration efficiency in the early training phase, and, in combination with dynamic gradient scaling, elastically adjusts gradient magnitude to balance gradient vanishing and the risk of explosion. In the middle and late stages of training, the adaptive learning rate compensation strategy suppresses validation accuracy fluctuations within 0.35% by dynamically adjusting the parameter update trajectory, ultimately achieving a 5.8% point improvement over the baseline Adam algorithm. This design effectively mitigates the oscillatory dispersion problem of traditional adaptive algorithms in regions of pathological curvature, providing a novel optimization paradigm for complex medical image modeling.

**Fig. 9:**
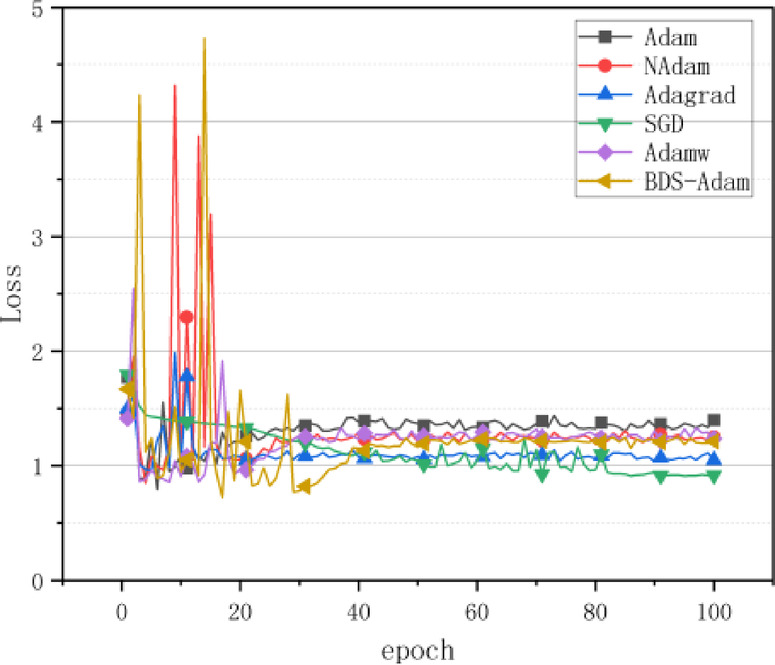
Comparison of loss values of various algorithms on Stomach dataset.

As shown in Fig. 9, the loss curves of different optimization algorithms on the Stomach dataset demonstrate distinct convergence patterns. Adam and NAdam consistently exhibit higher loss values throughout the training process, while BDS-Adam shows a rapid decline in the early stages, indicating superior initial convergence behavior. Although the final loss of BDS-Adam is not the lowest in this particular experiment, it maintains strong overall performance throughout the training. More importantly, as summarized in Tables 7 and 8, BDS-Adam achieves the lowest average loss and highest classification accuracy across multiple independent runs on both the CIFAR-10 and Stomach datasets. All improvements are statistically significant under the Bonferroni-adjusted threshold (*p* < 0.0001 in most cases), providing robust evidence of the consistent superiority of BDS-Adam compared to existing baselines.

To rigorously assess the performance advantages of BDS-Adam over baseline optimizers (SGD, Adagrad, AdamW, Adam, Nadam), we conducted statistical hypothesis testing based on five independent runs (*N* = 5) on both the CIFAR-10 and STOMCH datasets. We adopted a significance level of α = 0.05 and applied the Bonferroni correction to account for multiple comparisons, resulting in an adjusted threshold of α′ = 0.01.

**Table 7:** Mean accuracy (%) and significance vs. BDS-Adam on CIFAR-10 and STOMACH Datasets.

	Optimization Algorithm	Mean accuracy	Std Dev	Mean accuracy difference	vs. BDSAdam (*p*)
CIFAR-10	SGD	56.62%	1.23%	+ 30.17%	0.0000 √
	Adagrad	43.97%	0.39%	+ 42.82%	0.0000√
	Adamw	78.11%	0.77%	+ 8.68%	0.0000√
	Adam	78.03%	0.48%	+ 8.76%	0.0000√
	Nadam	77.61%	0.46%	+ 9.18%	0.0000√
	BDSAdam	86.79%	0.39%		
STOMCH	SGD	66.04%	2.02%	+ 11.08%	0.0004√
	Adagrad	65.00%	1.17%	+ 12.12%	0.0003√
	Adamw	65.56%	2.39%	+ 11.56%	0.0011√
	Adam	66.40%	1.55%	+ 10.72%	0.0004 √
	Nadam	66.80%	1.38%	+ 10.32%	0.0000√
	BDSAdam	77.12%	1.25%		

As summarized in Tables 7 (accuracy) and 8 (loss), BDS-Adam consistently outperforms all baseline methods with statistically significant margins. On the CIFAR-10 dataset, BDS-Adam achieved an average accuracy of 86.79% ± 0.39%, surpassing AdamW by + 8.68% with *p* < 0.0001. On the STOMCH dataset, it attained 77.12% ± 1.25%, exhibiting gains of + 10.32% to + 12.12% over all baselines, with *p* ≤ 0.0004.

In terms of loss, BDS-Adam achieved the lowest values across both benchmarks. On CIFAR-10, it obtained a loss of 0.7652 ± 0.0222, representing a 16.00% to 49.39% reduction compared to baselines. On STOMCH, the loss was reduced to 0.6909 ± 0.0447, yielding 30.0% to 43.4% relative reductions. All comparisons were statistically significant, with *p* < 0.0001 in 10 out of 12 cases and *p* ≤ 0.0004 in the remaining two, firmly surpassing the Bonferroni-adjusted threshold. These results provide strong statistical evidence that the performance improvements introduced by BDS-Adam are not due to random variation, but are instead attributable to its algorithmic innovations.

**Table 8:** Mean loss and significance vs. BDS-Adam on CIFAR-10 and STOMACH Datasets.

	Optimization Algorithm	Mean loss	Std Dev	Loss reduction	vs. BDSAdam (*p*)
CIFAR-10	SGD	1.1973	0.0273	36.09%	< 0.0001
	Adagrad	1.5120	0.0090	49.39%	< 0.0001
	Adamw	0.9109	0.0182	16.00%	< 0.0001
	Adam	0.9431	0.0446	18.86%	< 0.0001
	Nadam	0.9358	0.0503	18.23%	< 0.0001
	BDSAdam	0.7652	0.0222		
STOMCH	SGD	0.9862	0.0443	30.0%	< 0.0001
	Adagrad	1.0380	0.0231	33.4%	< 0.0001
	Adamw	1.2199	0.1203	43.4%	0.0002
	Adam	1.2042	0.1215	42.6%	< 0.0001
	Nadam	1.0928	0.0691	36.8%	< 0.0001
	BDSAdam	0.6909	0.0447		

Although BDS-Adam consistently improves classification performance across all three datasets, the degree of improvement varies significantly. This variation can be attributed to differences in dataset complexity, input dimensionality, and model capacity sensitivity. Specifically, MNIST is a relatively simple dataset composed of low-resolution grayscale images with limited intra-class variation. Most optimizers, including baseline Adam, already achieve high accuracy (> 98%) on this task, leaving little room for further improvement. Thus, the marginal 0.08% gain of BDS-Adam reflects a ceiling effect due to the dataset’s simplicity and the strong baseline performance.

In contrast, CIFAR-10 contains color images with more complex semantics and inter-class similarities, posing a greater challenge for optimization. BDS-Adam’s adaptive gradient smoothing and variance rectification mechanisms significantly enhance stability and feature learning in this context, leading to a substantial 9.27% accuracy gain over Adam. The Stomach dataset presents moderate difficulty, with high-resolution medical images and limited training data. BDS-Adam benefits from both gradient stabilization and fine-tuning adaptability (via pretrained MobileNetV2), achieving a 3.0% improvement. This result demonstrates the optimizer’s ability to handle noisy, small-scale, and domain-specific data effectively.

Overall, the results indicate that BDS-Adam’s advantages become more pronounced in datasets with higher complexity, richer gradients, or greater optimization instability, which aligns with its design goals.

## Data Availability

Data is provided within the manuscript：CIFAR10: [https://www.kaggle.com/datasets/gazu468/cifar10-classification-image](https:/www.kaggle.com/datasets/gazu468/cifar10-classification-image); Stomach: https://doi.org/10.1038/s41597-020-00622-y; Mnist: [https://www.cvmart.net/dataSets/detail/236](https:/www.cvmart.net/dataSets/detail/236).

## Declarations

### Availability of computer code and algorithm

wengshiqian/BDS-Adam: An Improved Adam Optimizer with Adaptive Variance Recti-fication and Borgesian Gradient-Aware Smoothing Dual Mechanisms.
